# Suture‐induced arthritis as a clinical mimicker of septic arthritis

**DOI:** 10.1002/ccr3.1318

**Published:** 2017-12-11

**Authors:** Nazuna Mizuno, Akira Mizuno

**Affiliations:** ^1^ Department of Infectious Diseases Otemachi Hospital 15‐1, Otemachi Kokurakita Kitakyushu Fukuoka 803‐8543 Japan; ^2^ 2‐4‐2 Kajimachi #201 Kokurakita Kitakyushu Fukuoka 802‐0004 Japan

**Keywords:** Differential diagnosis, foreign body arthritis, postoperative complication, septic arthritis

## Abstract

Foreign body‐induced arthritis is a rare etiology of mono‐arthritis. It should be suspected in the case of postoperative arthritis, even if some decades have passed since surgery. Histopathology of the tissues is required for a definitive diagnosis, and debridement is essential for complete recovery.

## Introduction

Making a differential diagnosis of arthritis and treating it are routine for infectious disease specialists. The major etiology of mono‐arthritis includes osteoarthritis, crystal‐induced arthritis, trauma, rheumatic diseases, and infection, and they frequently overlap. However, there is another etiology – foreign body‐induced arthritis – on which only a few studies have been reported. Here, we report a case of shoulder arthritis caused by surgical sutures.

## Case

An 84‐year‐old Japanese woman was brought to our emergency department. She had sustained a 40°C fever over 2 days. Intravenous ceftriaxone followed by oral levofloxacin was administered at the long‐term care facility. There were no remarkable findings on physical examination except for cognitive impairment and paralysis of the left upper and bilateral lower extremities caused by a previous cerebral infarction and by disuse syndrome.

Her medical history was significant for paroxysmal atrial fibrillation, cerebral infarction, Parkinson's disease, and dementia. She had been confined to bed in a nursing home and was practically without any mobility for several years. She had a surgical history of superior mesenteric artery thrombosis and a chronic subdural hematoma.

After urine and sputum samples and two sets of blood cultures had been submitted for laboratory testing, intravenous amoxicillin and clavulanic acid were started on suspicion of some bacterial infection with an unknown focus at that point. Nevertheless, she remained febrile, and all cultures turned out to be sterile. On the fifth day of admission, the patient was referred to our infectious disease department.

On physical examination, her temperature was 38.2°C, pulse 125 bpm, blood pressure 110/54 mmHg, and respiratory rate 20 breaths/min. The Glasgow Coma Scale was E4V4M3, which was typical for her. Her right shoulder was red, swollen, and inflamed. Her shoulder pain was aggravated by pressing the right acromioclavicular joint or by abduction of the right arm. The remainder of the examination was normal.

Laboratory tests 1 day before consultation revealed the following values: white blood cell count 4700/mL (reference range, 3500–9700) with a normal differential cell count, hemoglobin level 10.1 g/dL (reference range, 11.2–18.3) and platelet count 71,000/*μ*L (reference range, 140,000–397,000), aspartate aminotransferase level 38 U/L (reference range, 13–33), alanine aminotransferase level 23 U/L (reference range, 6–30), lactate dehydrogenase level 190 U/L (reference range, 119–229), and C‐reactive protein level 22.94 mg/dL (reference range, <0.2). Other parameters, including the levels of creatinine, blood urea nitrogen, and electrolytes, were within normal limits.

We aspirated 40 mL of yellow turbid synovial fluid from the right shoulder joint. The analysis showed 7765 cells/*μ*L (mostly polymorphonuclear neutrophils), a protein level of 3.0 g/dL, and glucose of 75 mg/dL. Radiography of the right shoulder showed osteoarthritic changes, and some metallic suture anchors in the greater and lesser tuberosities of the humerus, which indicated that she had undergone rotator cuff repair in the past. Magnetic resonance imaging showed massive fluid collection within the right glenohumeral joint with synovitis, showing an amorphous mass of intermediate signal intensity on T2‐weighted images. The surgically repaired rotator cuff was diffusely reruptured.

No bacteria were found in the fluid sediment by Gram and acid‐fast staining; however, we started intravenous piperacillin on suspicion of bacterial arthritis. While the temperature of the patient had decreased since the first fluid aspiration, frequent taps were required to control the arthralgia and fluid collection in the right shoulder joint. The cell count of the synovial fluid increased to 38,400 cells/*μ*L (88% polymorphonuclear leukocytes, 4% lymphocytes, and 8% monocytes), and a significantly elevated adenosine deaminase level (61.0 U/L) was observed on the third tap.

*Pseudomonas* species was isolated, but only from the initial synovial fluid culture. However, our hospital's microbiological facility was not able to distinguish between *Pseudomonas fluorescens* and *Pseudomonas putida*. Antibiotic sensitivity could not be identified because there were too few bacterial colonies and poor growth. We discontinued piperacillin use because her arthritis had not improved after 1 week of medication and three taps, and also because the initial synovial fluid cultures in blood culture bottles had been sterile.

**Figure 1:**
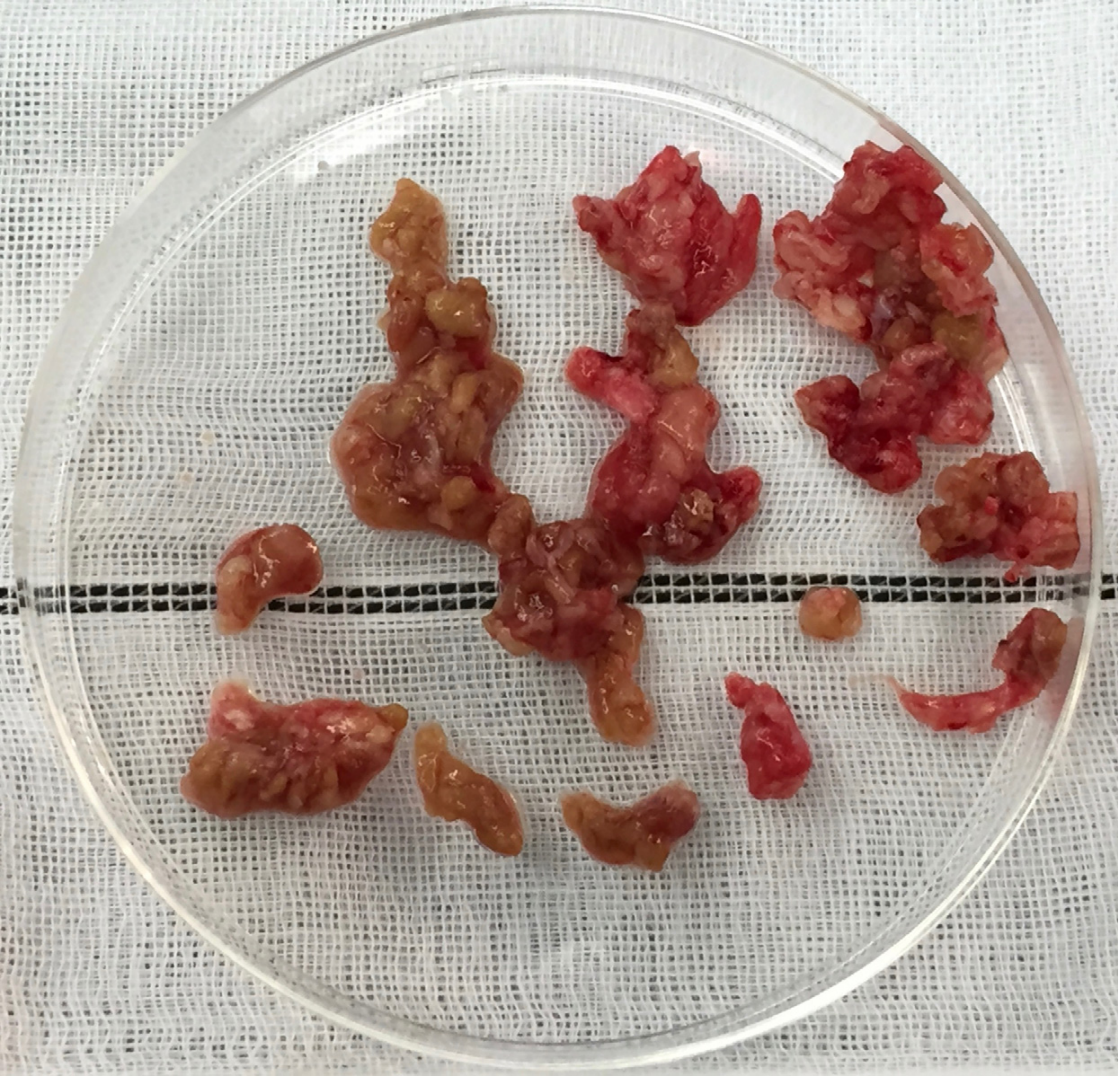
Hyperplastic synovial membrane with rice bodies.

**Figure 2:**
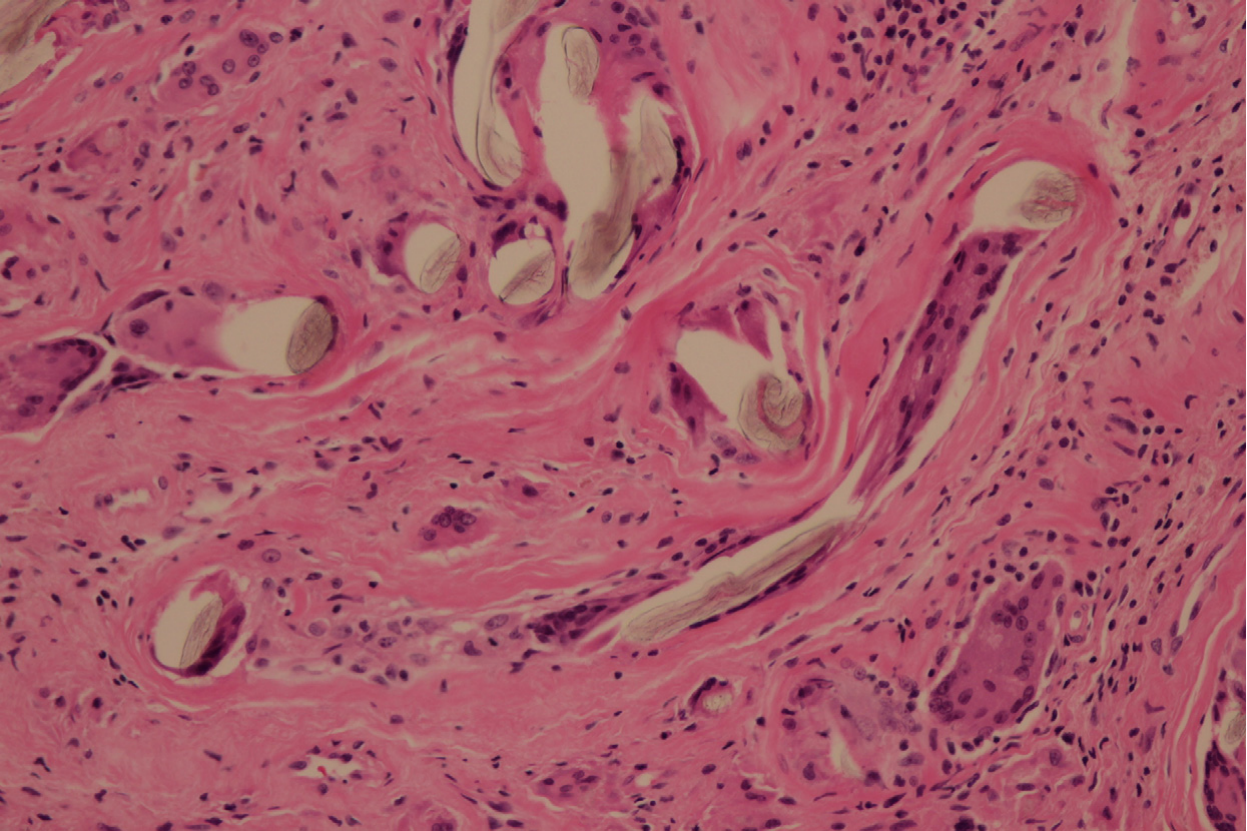
Inflammatory granulomas filled with numerous multinucleated giant cells which surrounded semitransparent and glossy surgical sutures.

Two weeks later, debridement and synovectomy of the right shoulder were performed to control the patient's culture‐negative nonresolving arthritis. Hyperplasia of the synovial membrane and the presence of rice bodies were observed during the operation (Fig. 1). Histopathology demonstrated inflammatory granulomas filled with numerous multinucleated giant cells, which surrounded semitransparent and glossy surgical sutures (Fig. 2). There were no pathological findings suggesting septic arthritis. Routine and acid‐fast bacilli smear and culture and polymerase chain reaction amplification for *Mycobacterium tuberculosis* were negative. Postoperatively, her persistent arthritis subsided completely, and the function of the right shoulder improved gradually.

## Discussion

Adverse human tissue reactions to artificial devices and materials have been acknowledged especially by surgeons and orthopedists as synthetics were first utilized for specific types of surgery 1. By contrast, foreign body‐induced arthritis is clinically uncommon, and only a few reports have been published. We found 36 patients diagnosed with such arthritis in 10 English‐language 2, 3, 4, 5, 6, 7, 8, 9, 10, 11 and two Japanese 12, 13 papers. Of these, 15 patients 2, 3, 4, 12, 13 were affected in large joints and 21 5, 6, 7, 8, 9, 10, 11 in small joints such as the carpometacarpal joint or temporomandibular joint. Arthritis usually occurred at various times (from 3 weeks to 8 years) after surgery. Pathologically proven foreign body reactions were observed in 27 cases, while the others were diagnosed clinically. Debridement was performed in all cases. Patients’ clinical outcomes after debridement were described for 22 cases. In 18 cases, the patients recovered fully, including our patient, and the other four complained of mild symptoms such as minimal swelling and pain during strenuous exercise.

We considered our patient to have suture‐induced arthritis for the following reasons: There was a pathologically obvious chronic reaction to the surgical sutures; tissue cultures were negative for pathogenic organisms; and the clinical course showed that the uncontrolled arthritis improved immediately after debridement. According to the surgical records on her right shoulder sent from the hospital where she had undergone an open rotator cuff repair 10 years previously, nylon, polyester, and polyethylene sutures were used.

On the other hand, our case was not typical of arthritis caused by a foreign body from three aspects. First, foreign bodies promoting inflammation basically localize exclusively to the implanted joint, and the disease progression is usually subacute or chronic. Acute arthritis with systemic symptoms mimicking septic arthritis – as in our case – is quite uncommon. Among 36 cases, only two patients 4 who had undergone total hip arthroplasty with Vicryl™ sutures presented with fever: one with a low‐grade fever (37.9°C) and the other with a high‐grade fever (39°C). Second, the timing of disease onset after surgery in our case was quite delayed. Even the longest case presented with arthritis of the knee 8 years after total knee arthroplasty 12. Particles of cobalt chromium alloy and polyethylene and bone cement were presumed to be the causal material in that case. Third, nonabsorbable sutures in our case led to arthritis even though such sutures give very low tissue reactions. From six cases 3, 4, 13 of suture‐related adverse reactions published so far, five were with Vicryl™ sutures, which are absorbable, and only one with FiberWire™ sutures, which are nonabsorbable. Other causal devices were as follows: eight following metal‐on‐metal total hip arthroplasty 2; five with Artelon™ spacers 5, 7, 8; eight with polyethylene mesh 6; five with Dacron™ grafts 10, 11; and three with Permacol™ grafts 9.

One hypothesis that could explain these three issues is that a bacterial infection with *Pseudomonas* species might have triggered arthritis in our case. At the outset, septic arthritis might have occurred, and frequent taps and antibiotics almost suppressed the inflammation but then initiated a foreign body reaction. In previous reports, only three patients had positive cultures, hemolytic *Streptococcus* in two cases 2 and nutritionally variant streptococci in one case 2, although the times after the index surgery and systemic symptoms were not clearly described. Thus, acute bacterial infection might induce or coexist with foreign body reactions.

There is a similar pathological condition in which artificial devices can cause adverse tissue reactions, namely Schloffer tumor. This is a rare inflammatory pseudotumor induced by nonabsorbable surgical sutures months to years postoperatively 14. Reactive fibrous granulomas with low virulent staphylococcal or streptococcal infections are the essential condition 14. Removing the foreign body and debridement are essential for treatment.

## Conclusions

This case shows that human tissues can react unexpectedly against artificial devices or material long after surgery. Such foreign body‐induced arthritis might be underdiagnosed among cases of aseptic synovitis, and it should thus be considered in patients with postoperative mono‐arthritis of which the result of the bacterial culture is equivocal or no crystal deposition. In these cases, debridement should be considered irrespective of the results of bacterial culture. Taking an adequate history of articular and periarticular surgery could be a clue to the correct diagnosis.

## Consent

Written informed consent was obtained from the patient's next of kin.

## Authorship

NM: drafted this manuscript. AM: revised and approved the final version.

## Conflict of Interest

The authors have no conflict of interest to declare.

## References

[1] Postlethwait, R. W. , D. A. Willigan , and A. W. Ulin . 1975 Human tissue reaction to sutures. Ann. Surg. 181:144–150. 122890 10.1097/00000658-197502000-00003 PMC1343743

[2] Judd, K. T. , and N. Noiseux . 2011 Concomitant infection and local metal reaction in patients undergoing revision of metal on metal total hip arthroplasty. Iowa Orthop. J. 31:59–63. 22096421 PMC3215115

[3] Pierannunzii, L. , A. Fossali , O. De Lucia , and A. Guarino . 2015 Suture‐related pseudoinfection after total hip arthroplasty. J. Orthop. Traumatol. 16:59–65. 24916148 10.1007/s10195-014-0300-4 PMC4348502

[4] Sayegh, S. , L. Bernard , R. Stern , J.‐C. Pache , I. Szalay , and P. Hoffmeyer . 2003 Suture granuloma mimicking infection following total hip arthroplasty. A report of three cases. J. Bone Joint Surg. Am. 85‐A:2006–2009. 10.2106/00004623-200310000-00023 14563812

[5] Robinson, P. M. , and L. T. Muir . 2011 Foreign body reaction associated with Artelon: report of three cases. J. Hand Surg. Am. 36:116–120. 21193131 10.1016/j.jhsa.2010.10.001

[6] Spaans, A. J. , E. J. M. L. van Heeswijk , D. E. Arnold , and A. Beumer . 2014 Foreign body reaction associated with polyethylene mesh interposition used for treatment of trapeziometacarpal osteoarthritis: report of 8 cases. J. Hand Surg. Am. 39:2016–2019. 25172386 10.1016/j.jhsa.2014.07.038

[7] Choung, E. W. , and V. Tan . 2008 Foreign‐body reaction to the Artelon CMC joint spacer: case report. J. Hand Surg. Am. 33:1617–1620. 18984346 10.1016/j.jhsa.2008.06.012

[8] Giuffrida, A. Y. , C. Gyuricza , G. Perino , and A. J. Weiland . 2009 Foreign body reaction to artelon spacer: case report. J. Hand Surg. Am. 34:1388–1392. 19801107 10.1016/j.jhsa.2009.05.006

[9] Belcher, H. J. , and R. Zic . 2001 Adverse effect of porcine collagen interposition after trapeziectomy: a comparative study. J. Hand Surg. Br. 26:159–164. 11281672 10.1054/jhsb.2001.0554

[10] Chantelot, C. , M. Rtaimate , and S. Chantelot‐Lahoude . 2004 Intracarpal synovitis related to Dacron interposition after trapeziectomy: a report of three cases. Chir. Main. 23:208–211. 15484683 10.1016/j.main.2004.05.007

[11] Acton, C. , G. Hoffman , H. McKenna , and F. Moloney . 1989 Silicone‐induced foreign‐body reaction after temporomandibular joint arthroplasty. Case report. Aust. Dent. J. 34:228–232. 2527491 10.1111/j.1834-7819.1989.tb00675.x

[12] Saisho, K. , H. Nagata , K. Maeda , K. Osamu , and K. Nabeshima . 2000 A case of bilateral revision total knee arthroplasty due to varus knee deformity associated with foreign body reaction in one knee and recurrent rheumatoid synovitis in the other. Jpn J. Rheumatol. Joint Surg. 18:213–217.

[13] Toru, Y. , M. Youichi , H. Junichi , K. Kazuhiko , O. Nobukazu , and I. Takashi . 2011 Glenohumeral osteoarthritis after arthroscopic bankart repair: a case report. Katakansetsu 35:1013–1016.

[14] Yazyi, F. J. , C. M. Canullan , N. F. Baglietto , R. F. Klappenbach , F. Alonso Quintas , J. Alvarez Rodriguez , and L. T. Chiappetta Porras . 2014 Schloffer's tumor: case report and review of the literature. Int. J. Surg. Case Rep. 5:1234–1237. 25437684 10.1016/j.ijscr.2014.10.044 PMC4275976

